# Time of sexual maturity and early egg quality of Japanese quails affected by in ovo injection of medicinal plants

**DOI:** 10.5194/aab-62-423-2019

**Published:** 2019-07-17

**Authors:** Karrar I. A. Al-Shammari, Justyna Batkowska, Kamil Drabik, Magdalena M. Gryzińska

**Affiliations:** 1 Department of Animal Production Techniques, Al-Musaib Technical College, Al-Furat Al-Awsat Technical University, Babylon Province, Babylon, Iraq; 2 Institute of Biological Basis of Animal Production, University of Life Sciences in Lublin, 13 Akademicka St., 20-950 Lublin, Poland

## Abstract

The aim of this study was to evaluate the time to sexual maturity and quality of initial eggs of Japanese quail affected by in ovo injection of plant extracts: ginger (GR), garlic (GC), oregano (O) and cinnamon (C). In total, 2400 eggs of Japanese quails were divided into six groups on the fifth day of incubation. Group I was the control group (NC), which was not injected. Other eggs were injected with 0.1 mL of liquid: group II – the positive control (PC) – with distilled water, group III with 1 % solution of GR, group IV with GC, group V with O and group VI with C. After hatching, the birds were reared in a cage system and fed with balanced mixtures, and 24 h lighting was used. The time at which birds reached sexual maturity was registered, while in the seventh week of rearing, 120 eggs were subject to quality evaluation. The traits of a whole egg (shape index as the ratio of egg width to egg length, weight, specific gravity), shell (strength, weight, thickness and density), albumen (weight, height), yolk (color, weight, index) were evaluated. At the earliest, on 36th day of life, eggs were laid by birds from the GC group, followed by C (37th day), O and NC (38th day), GR (39th day), and PC (41st day). During the first 2 weeks significantly more eggs were collected from the GC than from the other groups. The heaviest eggs derived from GC and GR groups, whereas the lightest came from the C group. Eggs from the GC group had the best shell strength and the greatest proportion of yolk. The use of medicinal herbs by injection in ovo may considerably modify both time of sexual maturity and quality of the initial eggs of Japanese quail.

## 1 Introduction

In ovo technology (IO) enables us to insert into an egg various substances that can modify the embryo development, as well as many physiological and production characteristics in the later life of the bird. Most often substances characterized by nutritional or immunostimulating modes of action are used. This last property may refer to plant extracts (PEs).

IO on different sites of the egg has been carried out routinely by many researchers (Maiorano et al., 2012) by some exogenous solutions, for example carbohydrates (Tako et al., 2004), proteins, amino acids (Ohta et al., 2001; Bhanja et al., 2005), vitamins (Al-Shammari, 2012), minerals, hormones, synbiotics or antibiotics. IO with aqueous PEs has two potential targets: firstly, as biological growth promoters, due to their contents of natural basic nutrients (proteins, carbohydrates, fat, vitamins, trace elements), which are regarded as primary ingredients of plants (Grashorn, 2010); secondly, as precautionary and antioxidant promoters, due to their biological EO (essential oil) contents and other bioactive components as plant secondary metabolites (Kabera et al., 2014).

Malheiros et al. (2012) recommended that in ovo administration with essential oils extracted from herbs (oregano, cinnamon, sage, rosemary) may have a positive effect on the increasing antioxidant status of hatched chicks. However, the injection of these oils in ovo is fraught with difficulties, basically because of their liposoluble nature. There is a lack of papers concerning this innovative topic regarding our tested PEs. The effect of different levels of PEs or their derivatives on hatching, productive and immune results through IO has been studied by researchers, who examined, for instance, α-galactoside of lupin seeds (Villaluenga et al., 2004), α-galactoside of pea (Pilarski et al., 2005), caffeine (McGruder et al., 2011), grape seed (Hajati et al., 2014), pollens of sunflower (Coşkun et al., 2014), wheat (Tako et al., 2014), garlic, tomato (Fazli et al., 2015), thyme, savory (Saki and Salary, 2015), or *Silybum marianum* (Morovata et al., 2016).

It is worth mentioning that some PEs were tested for their antiviral (Rezatofighi et al., 2014) and antimicrobial activity (Burt, 2004), as natural anticoccidial (Dalloul et al., 2006), as biological antioxidants reacting with free radicals (Wallace et al., 2010), for anti-inflammatory activity of the phytochemicals contained (Sahin et al., 2013) and for immunostimulant functions (Frankic et al., 2009).

The aim of this study was to evaluate the time to sexual maturity and quality of initial eggs of Japanese quail affected by in ovo injection of plant extracts: ginger (GR, *Zingiber officinale*), garlic (GC, *Allium sativum*), oregano (O, *Origanum vulgare*) and cinnamon (C, *Cinnamomum verum*). The choice of plants was determined by their various properties. It was hypothesized that PEs can have two positive effects on avian embryos: as natural growth promoters and as an antioxidant.

**Table 1:** Chemical composition of some active substances in powdered plant extracts.

Phytochemicals	GR	GC	O	C
Essential oil (%)	0.10	0.45	0.30	1.25
Flavonoids (%)	0.0035	0.0023	0.0023	0.007
O-dihydroxyphenols (%)	0.214	0.071	0.705	0.278
Valerenic acid (%)	0.0047	0.0128	–	–
Glucosinolates (µmol g${}^{-1}$)	0.031	–	0.004	–

GR – ginger; GC – garlic; O – oregano; C – cinnamon.

## 2 Material and methods

The material consisted of 2400 hatching eggs of Japanese quail. All eggs were numbered individually and allocated randomly into six groups before incubation, with 400 eggs per group (five replications in each). The research was conducted with the approval of the Second Local Ethical Committee (No. 16/2014), Poland.

**Figure 1:**
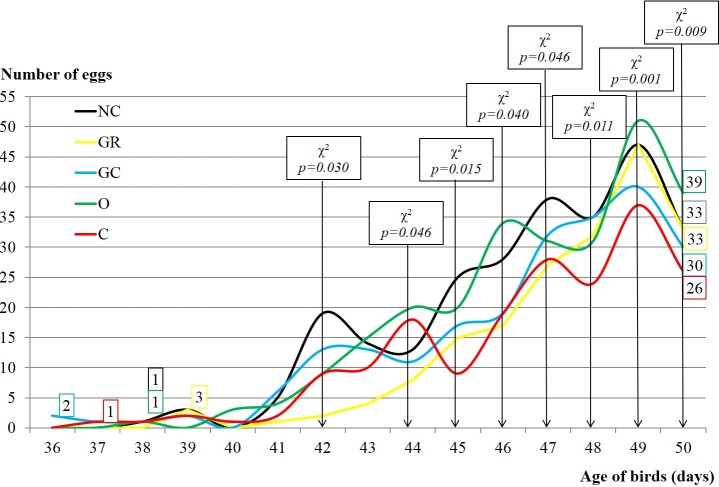
Sexual maturity and number of collected eggs of Japanese quails influenced by in ovo injection with aqueous solutions of plant extracts (200 per group with 5 replications). NC – negative control; GR – ginger; GC – garlic; O – oregano; C – cinnamon.

In a pre-experiment period, some of the fertile eggs were tested with an injection in ovo (IO) to an air cell with phenyl blue stain to determine whether the injected material diffused to all egg cavities throughout the egg (injection site). All phenyl blue stain was taken up by the injected egg, and, therefore, aqueous preparations of plant extracts (PEs) were implemented in this particular case. Commercial plant extracts were used (Bellako^®^); Table 1 presents the phytochemical content in the extracts chosen. The percentage of essential oil content in each plant was identified using a gas chromatography apparatus by the distillation method accredited for herbs and spice analysis in accordance with PN-A-79011-14:1998, PN-A-79097:2001 and PN – ISO 6571:2001. The distilled oil was expressed as mL per 100 g of dried powder (%). The sum of flavonoids was calculated on quercetin according to the spectrophotometric method accredited for plant material analysis, using a Shimadzu UV-1800 spectrophotometer apparatus. The determination sum of O-dihydroxyphenoles was calculated for caffeic acid (a method accredited for raw plant material) using a Dionex-ASE 150 accelerated solvent extractor and a Shimadzu UV-1800 spectrophotometer (Singleton and Rossi, 1965). A Dionex-ASE 150 accelerated solvent extractor, a SPD-M20A detector and a Shimadzu HPLC (high-performance liquid chromatography) system were used to determine the valerenic acid and glucosinolates. Moreover, the HPLC method was used to calculate the sum of glucosinolates on sinigrin, based on the dry-matter weight according to PN-ISO 10633-1:2000. Qualitative and semiquantitative analysis of essential oil as a percentage of individual components in the extract was determined by the GC/MS method (gas chromatography–mass spectrometry), and the GC/FID method (gas chromatography–flame ionization detector) was performed using a Varian Chrompack CP-3800 GC, equipped with a VF-5ms capillary column, a mass detector 4000 GC/MS/MS and a flame ionization detector (FID). The carrier gas was helium, at a flow rate of 0.5 mL min${}^{-1}$. Column temperature was initially 50 ${}^{\circ }$C for 15 min, then gradually increased to 250 ${}^{\circ }$C at a rate of 4 ${}^{\circ }$C min${}^{-1}$, and finally increased to 250 ${}^{\circ }$C at 10 ${}^{\circ }$C min${}^{-1}$. The procedure was performed with the use of a dispenser: 250 ${}^{\circ }$C, split 1:100. 1 µL of the solution was dispensed (10 µL or mg of the sample in 1000 µL of hexane). Kováts retention index was estimated on the basis of the n-alkane retention times (C10-C40). Mass spectral library, NIST Library and HP Chemstation was used. The composition of essential oil was expressed as grams of constituent per 100 g of essential oil (%).

A 1 % concentration solution of powdery PEs was prepared in distilled water. On the fifth day of incubation, eggs from each treatment were removed from the incubator to perform the IO technique. The air space of the egg was determined with a marker pen and disinfected with cotton swabs with 96 % ethanol, then manually holed from the top of the round end of the egg with a dental drill (10 000 RPM) to facilitate the injection of solutions at a maximum depth of 5 mm into the air cell by using a disposable tuberculin syringe (1 mL capacity). The experimental groups have been distributed as follows. Eggs from group I were not injected (NC). Because the PEs powder was dissolved in distilled water, group II (PC) was injected with only distilled water (0.1 mL) to determine whether there was any synergistic effect of manipulation. For IO in groups III, IV, V and VI the aqueous extracts of GR, GC, O and C were used with a dose of 0.1 mL each. The size of the dose had to be limited due to the small capacity of the air cell in the quail egg.

After IO the hole in egg shell was sealed with liquid paraffin by using a glass stirring rod. After the injection procedure, eggs were transferred to the incubator as soon as it was possible. The duration of manipulation time amounted to about 20 min; also all eggs from NC group were taken out from the setter for the same amount of time.

Eggs were hatched artificially using a BIOS hatching apparatus under standard conditions of incubation. Eggs in the setting compartment automatically turned 90${}^{\circ }$ every 3 h (eight times a day). On the 14th day of incubation the eggs were candled and moved from the setter to the hatching compartment. After 17.5 d of incubation, all hatched chicks were removed from each hatch basket. Each chick was individually marked with a numbered leg ring (up to the seventh day) and by wing tags (from the eighth day). All birds were kept under uniform management conditions throughout the experimental period in the following 7 weeks according to their treatments (four pens or groups). During 49 d of rearing, a balanced mixture specifically for meat-type quails was used. The birds were fed ad libitum. The ingredients and chemical compositions of the diets are presented in Table 2.

**Table 2:** Ingredients composition (%) and nutrient calculation of diet in the experiment.

Ingredients	%	Calculated chemical composition	
Corn	20.00	Crude protein (%)	22.722
Wheat	14.55	Metabolizable energy (Kcal kg${}^{-1}$)	2702.5
Wheat bran	15.00	Ether extract (%)	4.5590
Soybean meal (46 %)	27.00	Crude fibers (%)	5.4860
Sunflower meal (35%)	5.000	Crude ash (%)	6.9070
Soybean oil	2.250	Total calcium (%)	1.2430
Limestone	2.280	Available phosphorus (%)	0.3040
Salt (NaCl)	0.330	Sodium (%)	0.1890
DL-methionine (99 %)	0.050	Potassium (%)	0.9400
L-lysine (78 %)	0.040	Lysine (%)	1.1530
Protein + fat concentrate	1.000	Methionine (%)	0.3950
Premix*	0.500	Methionine + cysteine (%)	0.8160
Corngold^®^	12.00	Linoleic acid (%)	1.8210
Total	100.0	Vitamin A (IU kg${}^{-1}$)	10 000
		Vitamin D${}_{3}$ (IU kg${}^{-1}$)	2500.0
		Vitamin E (mg kg${}^{-1}$)	20 000

${}^{*}$ Premix (vitamin–mineral mixture) addition in diet used in two forms: premix 1 (up to 6 weeks of rearing) and premix 2 (in the seventh week of rearing). Premix 1 composition: vit. A – 2 000 000 IU kg${}^{-1}$; vit. D${}_{3}$ – 500 000 IU kg${}^{-1}$; vit. E – 4000 mg kg${}^{-1}$; vit. K${}_{3}$ – 400 mg kg${}^{-1}$; vit. B${}_{1}$ – 300 mg kg${}^{-1}$; vit. B${}_{2}$ – 900 mg kg${}^{-1}$; niacin – 4000 mg kg${}^{-1}$; pantothenic acid (vit. B${}_{5}$) – 2400 mg kg${}^{-1}$; vit. B${}_{6}$ – 600 mg kg${}^{-1}$; vit. B${}_{12}$ – 3000 µg kg${}^{-1}$; biotin – 20 000 µg kg${}^{-1}$; choline chloride – 50 000 mg kg${}^{-1}$; folic acid (vit. B${}_{9}$) – 160 mg kg${}^{-1}$; Fe – 10 000 mg kg${}^{-1}$; Mn – 12 000 mg kg${}^{-1}$; Cu – 2000 mg kg${}^{-1}$; Zn – 12 000 mg kg${}^{-1}$; I – 160 mg kg${}^{-1}$; Se – 40 mg kg${}^{-1}$; coccidiostat (monensin) – 20 000 mg kg${}^{-1}$. Premix 2 composition: vit. A – 2 000 000 IU kg${}^{-1}$; vit. D${}_{3}$ – 500 000 IU kg${}^{-1}$; vit. E – 4000 mg kg${}^{-1}$; vit. K${}_{3}$ – 400 mg kg${}^{-1}$; vit. B${}_{1}$ – 300 mg kg${}^{-1}$; vit. B${}_{2}$ – 900 mg kg${}^{-1}$; niacin – 4000 mg kg${}^{-1}$; pantothenic acid (vit. B${}_{5}$) – 2000 mg kg${}^{-1}$; vit. B${}_{6}$ – 600 mg kg${}^{-1}$; vit. B${}_{12}$ – 3000 µg kg${}^{-1}$; biotin – 20 000 µg kg${}^{-1}$; choline chloride – 50 000 mg kg${}^{-1}$; folic acid (vit. B${}_{9}$) – 160 mg kg${}^{-1}$; Fe – 10 000 mg kg${}^{-1}$; Mn – 16 000 mg kg${}^{-1}$; Cu – 2000 mg kg${}^{-1}$; Zn – 16 000 mg kg${}^{-1}$; I – 160 mg kg${}^{-1}$; Se – 40 mg kg${}^{-1}$.

The age of sexual maturity of females (200 per group with five replications each) was monitored by the laying of the first egg. Daily enumeration of total produced eggs until seven weeks was done. In the seventh week, a total of 120 eggs (24 eggs in each group) were collected to investigate their internal and external quality. The electronic set EQM (Egg Quality Measurements by TSS^®^) and Instron Mini 55 apparatus were used. The following egg traits were evaluated: egg weight, egg density or egg-specific gravity (calculated on the basis of egg weight measured in air and in water, Archimedes principle), egg shape index (calculated as the ratio of egg width to egg length), features describing shell (shell strength, shell weight, shell thickness and shell density calculated on the basis of egg weight and shell area), albumen (weight, height and Haugh unit), yolk (yolk color was determined with the 16-point scale by DSM^™^ yolk fan; yolk index as a ratio of the yolk height to its diameter; and yolk weight). A Vernier caliper was used to measure albumen height, the yolk index and the egg index.

The data were analyzed with the use of the statistical package SPSS 20.0PL (IBM Corp., 2011). The Kolmogorov–Smirnov test was carried out to ensure the normality of data distribution. The significance level was defined as 5 %. The obtained numerical data were verified by a t test and one- or two-factorial ANOVA and Tukey's test. The $\chi^{2}$ test was used to analyze the nonparametric data of experiment (i.e., mortality and hatchability).

## 3 Results

Due to the fact that the manipulation during the incubation (the IO) did not influence the evaluated traits, it was decided to omit the positive control group from further analysis.

Figure 1 shows time of sexual maturity achievement and the number of collected eggs, depending on particular groups. It was illustrated that females of GC and C achieved sexual maturity earlier than other groups, on 36th and 37th days post-hatch, respectively. Subsequently, females from the O, NC and GR groups reached sexual maturity on the 38th, 38th and 39th days. The greatest number of eggs up to the 50th day of birds' life was collected in the O group, the smallest in C (39 vs. 26). The number of eggs depended significantly on group from the 42nd until the 50th day of experiment.

**Table 3:** Egg quality of Japanese quails influenced by in ovo injection with aqueous solutions of plant extracts (at seventh week of birds' life, 24 eggs per group, 120 eggs in total).

Traits		Groups					SEM
		NC	GR	GC	O	C	
Whole egg	weight (g)	9.32${}^{\text{ab}}$	9.48${}^{\text{ab}}$	9.62${}^{\text{a}}$	9.29${}^{\text{ab}}$	8.78${}^{\text{b}}$	0.077
	density (g cm${}^{-3}$)	1.07	1.07	1.07	1.07	1.07	0.001
	index (%)	0.78	0.78	0.79	0.79	0.78	0.002
Yolk	weight (g)	2.54${}^{\text{ab}}$	2.68${}^{\text{ab}}$	2.83${}^{\text{a}}$	2.64${}^{\text{ab}}$	2.36${}^{\text{b}}$	0.036
	index (%)	0.45	0.46	0.45	0.46	0.48	0.005
	color (pts)	6.30${}^{\text{ab}}$	6.65${}^{\text{ab}}$	6.84${}^{\text{ab}}$	7.50${}^{\text{a}}$	5.89${}^{\text{b}}$	0.131
	proportion in EW (%)	27.29${}^{\text{ab}}$	28.27${}^{\text{ab}}$	29.30${}^{\text{a}}$	28.42${}^{\text{ab}}$	26.88${}^{\text{b}}$	0.243
Albumen	weight (g)	5.56	5.43	5.41	5.38	5.06	0.051
	height (mm)	4.51	4.30	5.04	4.40	5.06	0.114
	Haugh unit	90.09	89.71	93.79	90.62	94.45	0.567
	proportion in EW(%)	59.64${}^{\text{a}}$	57.27${}^{\text{ab}}$	56.29${}^{\text{b}}$	57.82${}^{\text{ab}}$	57.59${}^{\text{ab}}$	0.268
Shell	weight (g)	1.22${}^{\text{b}}$	1.37${}^{\text{a}}$	1.38${}^{\text{a}}$	1.27${}^{\text{a}}$	1.37${}^{\text{a}}$	0.019
	area (cm${}^{2}$)	21.17${}^{\text{ab}}$	21.42${}^{\text{ab}}$	21.60${}^{\text{a}}$	21.13${}^{\text{ab}}$	20.35${}^{\text{b}}$	0.116
	strength (N)	8.60	11.06	12.23	10.73	11.18	0.432
	thickness (mm)	0.139	0.142	0.149	0.185	0.177	0.007
	density (g cm${}^{-3}$)	4.19	4.61	4.35	4.20	3.83	0.082
	proportion in EW (%)	13.07${}^{\text{b}}$	14.46${}^{\text{ab}}$	14.41${}^{\text{ab}}$	14.49${}^{\text{ab}}$	15.53${}^{\text{a}}$	0.170

NC – negative control; GR – ginger; GC – garlic; O – oregano; C – cinnamon; EW – egg weight; SEM – standard error of mean; ${}^{\text{a, b}}$ means within rows (for groups) which differ significantly at p≤0.05.

Table 3 shows egg quality derived from various experimental groups. The biggest eggs were laid in the group injected with garlic extract (9.62 g), the smallest in the C group (8.78 g). A similar relation was found with regard to yolk weight and its proportion. Oregano extract caused the most intensive coloring of yolk. All injected groups demonstrated greater shell weight and proportions than in NC. An important result, though it is not statistically significant, seems to be the shell strength, which in the GC group is 30 % greater than in the NC group.

## 4 Discussion

It is well known that primordial germ cells (PGCs) (ancestors of oogonia), oogenesis process (the formation of new germ cells by mitotic divisions of oogonia) and the subsequent cytological changes in oocytes are associated with the development of the feminine ovary of avian species during incubation (Hong et al., 1995; Johnson et al., 2014). The process of oogenesis is completed by the time of hatching, and the ovaries of birds resemble those of eutherian mammals in that no new oogonia are created and proliferated by the germinal epithelium of the ovary after hatching. At the time of hatching, germ cells remain mostly in the diplotene stage of meiotic prophase I (called oocytes) and have begun to organize into primordial follicles (Johnson et al., 2014). During embryonic development in quail, Rong et al. (2011) observed that on day 4, there are lots of PGCs clustered in the region where gonads would be formed. Also, a few PGCs began to differentiate into oogonia on the 7th day and there was a further increase in oogonia number on the 10th and 11th days. Furthermore, an early original ovum and more original ova on the 13th and 14th days, respectively, are formed and distributed in the ovary, and the shape of the ovary tends to be mature and clear; more ova are found on the 17th day. Oogonia enter into a series of mitotic and meiotic divisions to form primary and secondary oocytes as well as the final mature ovum. Therefore, it may be that the positive manipulation by IO of birds during their embryonic life triggers early activation of ovary and oogenesis and leads the onset of sexual maturity occurring earlier, which is reflected in high egg production in the post-hatch period. Fazli et al. (2015) reported that the administration of GC and tomato extracts in ovo at day 5 influenced female ovary defeminization and sex reversal during the embryonic development stage. The essential compounds in GC and tomato are natural aromatase inhibitors (antiestrogen). Ovarian germ cells of quail embryos might have a substantial increase in antioxidative activity in the presence of PE solutions (Wallace et al., 2010). Moreover, all these dramatic changes might be reflected in increased shell weight and shell proportion traits, originating from females treated in their embryonic stage in all PE and C extract groups.

There are many factors which may have an impact on egg production and their quality. They range from the bird's genotype or age (Zita et al., 2009; Sarica et al., 2012) and feed additives (Safaa et al., 2008; Batkowska et al., 2018) to the system of rearing (Batkowska et al., 2017). However, in this case, it seems that differences in the number of eggs and their quality are secondary effects of sexual maturity. Egg production is strongly influenced by the start of pullet photostimulation (Silverside et., 2006). Birds which start to lay eggs earlier produce them more than females which achieve maturity later. Also, the first eggs from younger birds may be heavier (Nassar et al., 2017). At the same time, typically bigger eggs contain more albumen and a smaller proportion of yolk, which is negatively related to egg size (Johnston and Gous, 2007), but attention should be paid also to the bird species because the proportions of the main egg elements differ among them (Song et al., 2000; Dudusola, 2010; Batkowska et al., 2017). Also, it is likely that the PEs used may affect egg traits. Garlic additive contributes to increasing Haugh's units and to decreasing shell weight (Canogullari et al., 2009); also, it may improve shell strength (Lokaewmanee et al., 2014). Ginger essential oil significantly increases the egg weight of Japanese quail; however, its effect depends strictly on the dose (Tchoffo et al., 2017). Some authors suggest (Arpášová et al., 2013) that the egg production and all qualitative parameters of egg yolk are not significantly influenced by oregano oil addition. Others found (Christaki et al., 2011) that oregano leaf additive increases the intensity of yolk color redness ($L^{*}a^{*}b^{*}$ scale) in quail eggs. In our study the darkest yolks also derived from the O group. The cinnamon may improve egg shell weight and its thickness (Vali and Mottaghi, 2016) as well as increase the egg mineral content (iron, Zn, and copper) compared to that in the control group of quails (Vali et al., 2013). It was proved that the mixture of powdered herbs (garlic, cinnamon, yarrow, rosemary, thyme, basil, oregano) considerably influences the physiological and production parameters of laying hens as well (Gerzilov et al., 2015).

It should be emphasized that most of references describing the influence of PEs on egg quality are based on nutritional experiments, in which birds were fed with extract additives. The impact of PEs injected in ovo have been not described. However, if the effect of PEs used in ovo was restricted to the age of sexual maturity of birds, it results rather from modification to the developing embryo's physiology than the nutritive value, and this phenomenon requires further detailed research.

## 5 Conclusions

Females from groups injected with garlic and cinnamon extracts achieved sexual maturity earlier than those in other groups; however, the greatest numbers of eggs were laid by quails from the oregano group. The extracts of ginger, garlic, oregano and cinnamon given in ovo visible influenced the quality traits of the eggs obtained, but the mode of action of a particular extract seems to be different. From an economical point of view the heaviest eggs with the thickest shell were found in the group injected with garlic extract, but for the consumer, the most intensive yolk color was found in eggs from quails injected with oregano. The phenomenon of the influence of the in ovo injection procedure on such late post-hatch evaluated traits needs further analysis.

## Data Availability

The original data are available upon request from the corresponding author.
